# Releasing the Lockdown: An Emerging Role for the Ubiquitin-Proteasome System in the Breakdown of Transient Protein Inclusions

**DOI:** 10.3390/biom10081168

**Published:** 2020-08-10

**Authors:** Yuval Reiss, Elisheva Gur, Tommer Ravid

**Affiliations:** Department of Biological Chemistry, The Hebrew University of Jerusalem, Jerusalem 91904, Israel; elisheva.gur@mail.huji.ac.il

**Keywords:** protein quality control, the ubiquitin-proteasome system, chaperones, misfolding, refolding, disaggregation, degradation

## Abstract

Intracellular protein inclusions are diverse cellular entities with distinct biological properties. They vary in their protein content, sequestration sites, physiological function, conditions for their generation, and turnover rates. Major distinctions have been recognized between stationary amyloids and dynamic, misfolded protein deposits. The former being a dead end for irreversibly misfolded proteins, hence, cleared predominantly by autophagy, while the latter consists of a protein-quality control mechanism, important for cell endurance, where proteins are sequestered during proteotoxic stress and resolved upon its relief. Accordingly, the disaggregation of transient inclusions is a regulated process consisting of protein solubilization, followed by a triage step to either refolding or to ubiquitin-mediated degradation. Recent studies have demonstrated an indispensable role in disaggregation for components of the chaperone and the ubiquitin–proteasome systems. These include heat-shock chaperones of the 40/70/100 kDa families, the proteasome, proteasome substrate shuttling factors, and deubiquitylating enzymes. Thus, a functional link has been established between the chaperone machinery that extracts proteins from transient deposits and 26S proteasome-dependent disaggregation, indicative of a coordinated process. In this review, we discuss data emanating from these important studies and subsequently consolidate the information in the form of a working model for the disaggregation mechanism.

## 1. Introduction

Alois Alzheimer’s discovery in 1906 of senile plaques in histological brain sections [1] attracted little attention from the scientific community [2]. Nevertheless, more than a hundred years later, the discovery of Alzheimer’s disease, as well as other proteinopathies, has initiated a major scientific endeavor to diagnose and mitigate their debilitating consequences. It also led to the realization that intracellular inclusion bodies are biochemically and physiologically heterogeneous and that some of these depositions are not merely a protein graveyard but rather, dynamic bodies that serve a vital physiological function. In this review, we shall discuss the turnover of dynamic protein inclusions, particularly the manner of their dissolution, focusing on the role of the ubiquitin-proteasome system (UPS) in this process.

**Figure 1:**
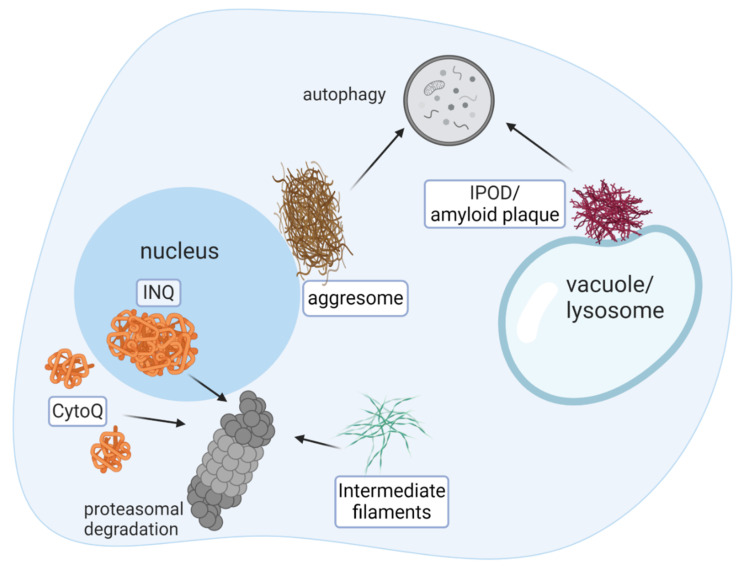
Misfolded proteins are sequestered in distinct cellular deposition compartments. Cellular inclusions are discerned by their morphology, protein composition, and deposition sites. Clearance of immobile deposits, such as aggresome, IPOD, and other mature amyloid inclusions is carried out predominantly by autophagy, while, transient-QC bodies, composed of intermediate filament, Cyto-Q and INQ are degraded by the 26S proteasome. Nearly all inclusions (except for aggresomes) are conserved in evolution, from yeast to human. The principles of the inclusion clearance mechanism are conserved as well.

More than two decades ago, while investigating the endoplasmic reticulum-associated degradation of Cystic Fibrosis Transmembrane conductance Regulator (CFTR) in human embryonic kidney 293 (HEK) cells, R. Kopito and colleagues discovered that upon proteasome inhibition, the protein accumulates in cellular inclusions [3]. These inclusions, coined “Aggresomes”, are localized to a perinuclear region and the cell periphery and are eventually cleared by ubiquitin-dependent autophagy [4,5]. A decade later, two types of intracellular inclusions were identified in the yeast *Saccharomyces cerevisiae* and in mammals, that were discerned according to their localization, mobility, and protein content [6]. Consequently, the inclusions were dubbed “insoluble protein deposit” (IPOD) and “Juxtanuclear protein control compartment” (JUNQ). Further characterization of JUNQ discerned it as an Intra Nuclear Quality control body, termed herein as “INQ” [7]. Concomitantly, another form of inclusions, composed of highly dynamic misfolded protein deposits, was identified in yeast and named Q-bodies or CytoQ [7,8]. Multiple small CytoQ form during proteotoxic stress and subsequently merge into one or two inclusions, apparently the INQ [7,8], that are ultimately cleared by the proteasome. The dynamics of CytoQ is strictly ATP-dependent and most importantly, their formation improves cell fitness during proteotoxic stress [8]. Subsequently, it has been proposed that CytoQ/INQ formation is a protein quality control (PQC) mechanism that quarantine misfolded proteins during proteotoxic stress and that a failure to refold or to degrade them results in the accumulation of large, apparently deleterious, inclusions [8]. Thus, the heterogeneity of cellular inclusions, discerned by characterization of their morphology, composition, dynamics, and cellular deposition sites [9] has been established (Figure 1).

Various disordered proteins can form amyloid inclusions, the hallmark of human neurodegenerative diseases, such as familial Alzheimer’s, Parkinson’s, and ALS, among others [10,11]. In several hereditary neurodegenerative diseases, the seed for protein inclusion body formation is the intermolecular interaction between molecules of a single, aberrant protein [12]. However, in sporadic, aging-related proteinopathies, where a genetic cause cannot be identified, immobile amyloid inclusions contain a variety of proteins that have undergone irreversible unfolding and accumulate due to deterioration of cellular PQC networks [13,14]. In contrast to amyloid inclusions, dynamic protein deposits are continuously generated and dissolved but tend to accumulate during proteotoxic stress [8]. Hence, this type of transient quality control inclusions is herein collectively termed “transient-QC” bodies. The fact that both the sequestration and clearance of transient-QC bodies are spatially and temporally regulated, highlights the critical importance of transient protein sequestration for the management of protein homeostasis [9,15].

The removal of irreversible aggregates, such as the yeast IPOD and other amyloid inclusions in higher eukaryotes, is primarily carried out by ubiquitin-dependent autophagy (Figure 1), a process mediated by adaptor proteins that bind ubiquitylated autophagy substrates and the phagosome receptor Atg8/LC3 [16,17]. Conversely, early, premature amyloid inclusions and transient-QC deposits are cleared by a mechanism termed disaggregation, which is essentially the extraction of proteins from insoluble aggregates. While autophagy permanently and indiscriminately disposes of IPOD content, disaggregation entails diverting liberated proteins to either refolding or degradation by the 26S proteasome (Figure 1). Furthermore, in contrast to autophagy, selective disaggregation appears to require the removal of polyubiquitin chains, as suggested by the critical role of deubiquitylation (DUB) enzymes in the process [18]. Additional findings demonstrating that autophagy kicks in to dispose of soluble misfolded protein aggregates when the proteasome is inhibited or overwhelmed, indicate the existence of crosstalk between these two degradation systems [19,20,21].

Despite expanding knowledge about the biology of protein deposits and the recognition that they are tightly controlled, highly conserved, PQC compartments that quarantine defective proteins, much remains vague. Overwhelming of the chaperone system is likely a trigger for misfolded protein deposition when Hsp70-dependent delivery of ubiquitylated proteins to the proteasome is inhibited, leading to their sequestration [22,23]. It is still unclear how the relief of proteotoxic stress triggers aggregate clearance and how the ensuing fate of disaggregated proteins to either refolding or proteasomal degradation is determined. Recent findings implicating the ubiquitin system in the regulation of misfolded protein deposition and specifically, in their breakdown, may hold answers to these questions. Here we review new evidence for the role of chaperones and the UPS in the context of a putative regulated mechanism that selectively releases proteins, determines their folding state, and consequently, diverts them to the appropriate pathway. We further integrate these findings into a general working model that offers future research directions.

## 2. Misfolded Proteins Are Extracted from Cell Inclusions and Sorted by Molecular Chaperones

The term “triage” in PQC was initially coined by Gottesman, Wickner, and Muarizi, referring to a bacterial chaperone mechanism that discerns the folding state of proteins and subsequently targets misfolded proteins to degradation [24]. It is most likely a selective process whereupon initially, individual proteins are extracted and solubilized, after which their folding state is discerned and accordingly, refolding or disposal is determined. When a protein is wedged in the compressed protein environment of the inclusion, its folding dynamics is affected by inter protein contacts, therefore, determination of a protein’s folding state likely requires prior solubilization. Thus, during inclusion body dissociation, proteins must be extracted and triaged to either refolding or degradation. We herein refer to disaggregation as a coupled solubilization-triage process.

Extraction of proteins from cellular inclusions requires the combined function of Hsp/Hsc 70 family members, their Hsp40 co-chaperones (J-domain proteins), and Hsp110 nucleotide exchange factors (NEFs) [25]. In general, the disaggregase constituents are modular, enabling functional versatility in terms of binding and recruitment of folding and degradation factors. Hsp70s carry out the extrusion while Hsp40 and Hsp110 chaperones ensure energy availability and delivery by activating ATP hydrolysis and ADP/ATP exchange, respectively. In yeast, these chaperones cooperate with Hsp104, an AAA+ ATPase hexamer that provides the energy for the extraction [26,27]. Intriguingly, the human disaggregase machinery disassembles protein aggregates independently of Hsp100 family members, through the cooperation of mixed class J-domain proteins, Hsp70s, and NEFs [28,29,30].

A recent study from the late Stephan Jentsch lab [31] has demonstrated that the yeast Hsp70/Apj1 bi-chaperone pair, in cooperation with Hsp110, exclusively couples the dissolution of INQ to 26S proteasome-mediated degradation, independently of Hsp104 function [32]. The involvement of the Hsp40 co-chaperone in the proteasome-dependent disaggregation of transient-QC is in line with findings that established Hsp40 co-chaperones not only as Hsp70 activators but also as regulators of their binding specificity and functional diversity [32,33,34,35]. Now, the den Brave study, not only establishes the function of the nuclear Hsp40, Apj1, in INQ clearance, but it also indicates that Hsp104-dependent refolding and proteasome-mediated degradation of solubilized misfolded proteins are independent disaggregation triage reactions [32]. Yet, Hsp104 activity is required for the turnover of aggregation-prone, membrane-embedded, Endoplasmic Reticulum-Associated Degradation (ERAD) substrates, but not for their soluble counterparts [36]. These findings indicate that yeast Hsp104 is capable of contributing an extraction force for both the refolding of proteins that emerge from trans-Q and the retro-translocation of ERAD substrates across the ER membrane.

## 3. A Role of the Ubiquitin-Proteasome System in Protein Quality-Control Triage Decisions

Unfortunately, like in real life, where not every ailment can be cured, terminal misfolding results in afflicted proteins being eliminated by proteolysis. This is where the UPS plays a lead role. The findings that overexpression of mutant CFTR or inhibition of the proteasome in cultured mammalian cells results in aggresomes formation [3,37], led to the understanding that proteasomes play a key role in the dynamics of protein inclusion bodies. Yet, it was only when the dynamics of pre-formed protein inclusions in yeast had been investigated under conditions where new protein synthesis is curtailed, that a key role of proteasomes in transient-QC clearance was realized [8,32,38].

### 3.1. Proteasome Inhibition Induces Transient-QC Accumulation

Protein inclusion levels increase when ubiquitin-mediated degradation of misfolded proteins is compromised, suggesting that the 26S proteasome function is critical for the maintenance of transient-QC bodies. Studies of the dynamics of pre-formed heat stress-induced inclusions in proteasome-inhibited yeast cells have revealed that proteasomes function is dispensable for CytoQ formation and subsequent merger to larger depositions [5,8]. However, proteasome inhibition prevented the dissolution of misfolded protein depositions upon recovery from heat stress, indicating that it primarily functions during disaggregation [8].

While the essential role of the proteasome in transient-QC disaggregation has been established [8,18,32], one should take into account that these studies often follow the fate of proteins that are beyond refolding and invariably degraded by the proteasome. Therefore, in each case, aggregate dissolution depends on proteasome activity. It is still unclear what proportion of protein, released from authentic transient-QC bodies under normal and proteotoxic stress conditions, are folded rather than degraded and how the initial folding state is resolved.

### 3.2. Proteasomes Are Associated with Transient-QC Deposits

During aging, a substantial proportion of proteasomes appear to be associated with protein deposition sites [39]. Apparently, they become available for UPS-mediated degradation only when the cellular protein folding capacity is increased, as evident by the observation that overexpression of Hsp104 in aged yeast cells increases UPS-mediated degradation without affecting the expression of its components [39]. This finding implies that folding has precedent over degradation. Nevertheless, when initial folding attempts are futile, degradation ensues. Most importantly, the fact that disaggregation is attenuated when either the proteasome or Hsp104 are inhibited [8,33], indicates that extraction is coupled to misfolded protein triage, apparently the rate-limiting step of disaggregation.

Direct evidence for the mobilization of mammalian proteasomes to protein deposition sites was provided by in situ Cryo-EM studies, using poly-GA-containing sequences that correspond to Neuronal poly-GA aggregates in amyotrophic lateral sclerosis and frontotemporal dementia [40]. Notably, proteasomes were not observed in association with polyQ-bodies composed of expanded huntingtin exon 1 aggregates [41], indicating that aggregation substrates determine the chemical as well as the biological properties of the inclusion. Proteasomes co-localized with poly-GA aggregates are stalled in an unproductive conformation and therefore their recruitment may hamper proteostasis [39]. A possible explanation for the inactivation mechanism was proposed, based on in vitro studies, where neurodegeneration-derived protein oligomers inhibited 26S proteasome activity by allosterically blocking substrate entry into the 20S catalytic core [42]. Thus, by sequestering inactive proteasomes, amyloid aggregates reduce the cellular proteolytic capacity and exasperate proteotoxic stress. However, catalytically inactive proteasomes can still operate in disaggregation by disassembling large fibrils composed of recombinant proteins, into smaller assemblies [43]. This function is attributed to the ATP-dependent, proteolysis-independent, activity of the proteasome holoenzyme. The association of proteasomes with inclusion bodies is also observed in plants where GFP-tagged 26S proteasomes transiently associated with cytoplasmic foci during recovery from heat stress [44]. These heat-induced inclusions are extensively ubiquitylated, implying that both the proteasome and ubiquitin conjugates play a role in disaggregation.

### 3.3. UBQLN2 Bridges between Transient-QC Bodies and the 26S Proteasome

The elimination of transient-QC bodies entails preliminary solubilization of monomers, only after which, the liberated misfolded proteins are subjected to proteasomal degradation [32,38,45,46]. Understanding how solubilized misfolded proteins that fail to refold are targeted to the proteasome is key to elucidating the operation of the PQC network in health and disease.

A seminal study by the Kurz lab in mammalian cells implicates the proteasome shuttle factor Ubiquilin-2 (UBQN2) in proteasome-mediated elimination of transient-QC [38]. Both human UBQLN2 and its yeast ortholog Dsk2 bind to Lys48-linked poly-ubiquitin chains via the C-terminal UBA domain, while the N-terminal UBL domain interacts with the proteasome, thus intermediating protein degradation [47,48,49,50,51]. The Kurz study shows an association of UBQLN2 with inclusions in mice brains, while co-immunoprecipitation experiments reveal direct UBQLN2 association with Hsp70s. Importantly, the abolishment of UBQLN2 binding to the Hsp70 impairs the breakdown of heat shock-induced inclusions. Thus, UBQLN2 emerges as a critical factor for the formation of UBQLN2/Hsp70/proteasome ternary triage complex, apparently acting as an adaptor between proteasomes and the Hsp70 component of the disaggregase via its stress-induced protein 1 (STI-1) and ubiquitin-like (UBL) domains, respectively. In addition to STI-1, the UBA domain of UBQLN2 is also essential for the association of proteasomes with heat-induced inclusions, suggesting that UBQLN2 is initially mobilized to aggregates by Hsp70 whereupon it binds ubiquitin-conjugated proteins. Noteworthy is the dual function of the UBL domain: Not only does it bind the 26S proteasome, but it also activates it [52]. Thus, in addition to bridging between the disaggregases and proteasomes, UBQLN2 may also promote disaggregation by enhancing the degradation of solubilized protein.

### 3.4. Deubiquitylation Activity is Required for Transient-QC Clearance

When the Hsp70 capacity is limiting, such as during prolonged proteotoxic stress, cells sequester misfolded proteins in a distinct transient-QC nuclear hub (INQ). Ubiquitin conjugation may drive misfolded protein sequestration into transient-QC [23,53,54]. The finding that the amount of insoluble poly-ubiquitin conjugates is substantially increased following heat stress, suggests that the sequestered proteins retain their ubiquitin moiety [38]. Consequently, to better understand the role of proteasome-mediate degradation in selective disaggregation one must consider how the ubiquitylation state affects transient-QC solubilization and triage. A couple of arguments assert that ubiquitin conjugates must be removed during triage: 1. Ubiquitin conjugation influences protein folding dynamics, thwarting the possibility of appropriate refolding [55]. 2. Ubiquitin conjugation would inevitably target solubilized proteins to the proteolytic pathway, regardless if they are re-foldable. 3. The penetration of a degradation substrate into the 20S proteolytic chamber requires prior removal of the ubiquitin moiety. Findings that yeast Ubp3 DUB activity enhances transient-QC turnover [18], further support the proposition that the removal of ubiquitin chains is required for misfolded protein disaggregation.

The yeast DUB enzyme, Ubp3, initially cloned by the A. Varshavsky Lab [56], participates in various cellular reactions, including gene silencing, aneuploidy, osmotic stress, and vesicular trafficking [57]. In regards to proteolysis, deletion and overexpression of Ubp3 attenuates and stimulates, respectively, ubiquitin-mediated degradation by the removal of polyubiquitin from degradation substrates at the 26S proteasome [58,59,60], thereby facilitating unfolding and threading of the proteolysis substrate into the core of the catalytic 20S complex [61]. A role for Ubp3 in PQC was initially demonstrated by Baxter and Craig who found that overexpression of the enzyme suppresses the temperature sensitivity of a mutant yeast strain, devoid of the two key Hsp70 chaperones, *SSA1* and *SSA2* [62]. Obviously, in a heat-stressed environment, where Hsp70 is limiting, the load of misfolded proteins that are targeted to PQC-associated ubiquitylation increases substantially. Hsp70s function is necessary for protein folding, degradation, and transient-QC turnover [63,64,65]. Consequently, during proteotoxic stress, their shortage is exasperated, resulting in a massive buildup of inclusion bodies [65]. Now, an intriguing study from the Nystrom Lab, conducted in yeast, provides an explanation for the suppression of the Hsp70 requirement and the consequent growth advantage conferred by Ubp3 overexpression [18]. The study shows that upon recovery from heat shock, *ubp3∆* cells display delayed clearance of INQ and accumulate age-related inclusion bodies. In reciprocal experiments, overexpression of Ubp3 reduces aggregation and cytotoxicity of inclusions harboring a temperature-sensitive mutant protein in *ssa1/ssa2∆* cells. The turnover of the heat-induced protein depositions is strictly dependent on the Ubp3 DUB activity and most importantly, it requires the activity of stress response elements, primarily UPS components, as is evident by the inability of Ubp3 overexpression to enhance inclusion solubilization in cells deleted of the stress-regulated transcription activator Rpn4 [18]. The fact that Ubp3 DUB activity can overcome Hsp70 deficiency, suggests that the removal of poly-ubiquitin conjugates promotes misfolded protein solubility, and is in perfect agreement with the findings that poly-ubiquitin chains promote misfolded protein sequestration [23].

## 4. A Putative Model for the Mechanism of Triage Decisions during Disaggregation

**Figure 2:**
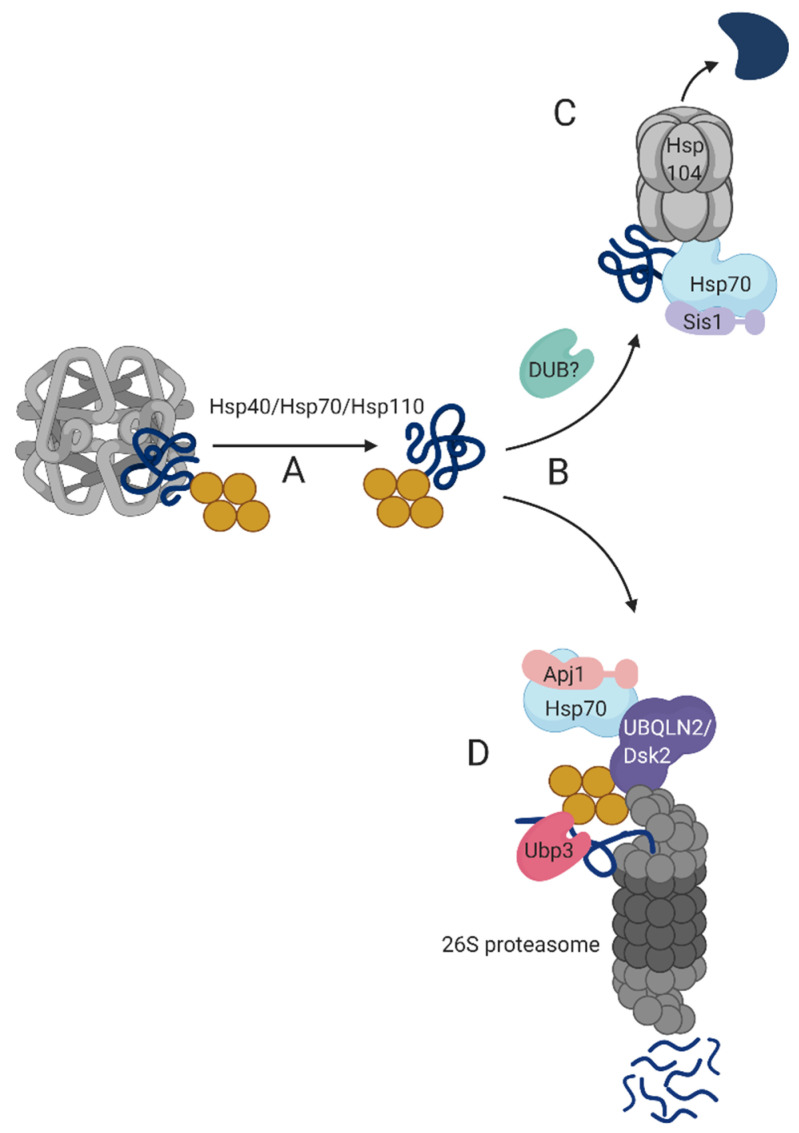
Working model of transient-QC disaggregation. (**A**) Misfolded proteins in transient-QC are dissolved by Hsp40/Hsp70/Hsp110 chaperones. (**B**) Once solubilized, misfolded proteins are sorted to either (**C**) refolding or (**D**) degradation. Protein refolding requires the cooperative activity of Hsp40/Hsp70 bi-chaperones and Hsp104. Protein degradation is facilitated by adaptor proteins, such as UBQLN2 that bridge between the ubiquitylated substrate and the proteasome 19S regulatory subunit. Ubp3 and possibly additional DUBs remove poly-ubiquitin chains from solubilized misfolded proteins prior to proteolysis and refolding. Yellow, ubiquitin conjugates; Dark blue, substrate.

Much progress has been made during the last decade towards elucidating the physiology and biology of transient protein deposits. It is now evident that misfolded proteins are subjected to multiple fates, depending on their intrinsic folding state, post-translational modification(s), and cellular environment. Traditionally, studies of protein sequestration dynamics have focused on factors that trigger inclusion body formation. The current review has assembled the recent advances made in the investigation of aggregate breakdown with emphasis on the role of the UPS. We now consolidate this data into a model for the disaggregation mechanism of transient-QC bodies (Figure 2).

The turnover of transient-QC is determined by the rates of misfolded protein coalescence and solubilization. Under normal physiological conditions, high protein folding capacity and efficient UPS activity determine a rapid turnover of transient-QC, hence, they are practically invisible. Enhanced sequestration, concomitant with reduced disaggregation and misfolded protein degradation during proteotoxic stress, increases coalescence and decreases disaggregation and degradation rates, lowering transient-QC turnover. As a result, transient-QC bodies accumulate in nuclear and/or juxtanuclear sites.

Transient-QC bodies disaggregation entails several consecutive steps: (A) Initially, sequestered proteins are extracted by a disaggregase machinery, composed of an Hsp70/Hsc70 that cooperates with alternate Hsp40s and Hsp110. (B) Once sequestered proteins are solubilized, they are subjected to a triage, carried out by either (C) Hsp104 foldase in yeast or a metazoan functional homolog or, (D) the 26S proteasome. The Hsp40 co-chaperone determines the triage path: Hsp70/Apj1 determines 26S proteasome-mediated degradation while the Hsp70/Sis1pair facilitates folding [32].

Proteasome-mediated degradation of transient-QC- solubilized misfolded proteins requires specialized auxiliary proteins, such as mammalian UBQLN2 (and possibly its yeast ortholog, Dsk2) and yeast Ubp3. UBQLN2 is recruited to transient-QC bodies via Hsp70 where it binds ubiquitylated proteins through its UBA domain. The UBL domain of UBQLN2 binds proteasomes, thus facilitating proteasome sequestration in the vicinity of transient-QC and consequent coupling of degradation to disaggregation. The proteasome-associated Ubp3 DUB activity is required for proteasome-dependent degradation of solubilized transient-QC proteins [18]. Whether Ubp3 operates before UBQLN2/Dsk2 association or after the shuttle factor has delivered its cargo to the proteasome is yet to be determined. Solubilized proteins destined to refolding are possibly deubiquitylated and presented to the Hsp104 foldase via a Hsp70/Sis1 bi chaperone that likely also contribute to the refolding process.

## 5. Future Perspectives

Multiple studies in yeast during the last decade, primarily from the Bukau, Nystrom, and Frydman labs, established a detrimental function for disaggregation in transient-QC turnover and cell fitness. Further studies in mammalian cell systems indicate that disaggregation is an evolutionarily conserved mechanism. Yet, a substantial research effort is still necessary to better understand the relative contribution of disaggregation to the turnover of protein inclusions. Understanding aggregation dynamics begs to answer key questions such as: What triggers aggregation and whether it is induced by default when chaperone availability becomes scarce, or rather, a tightly regulated process. The mechanism by which the PQC system discerns the refolding potential of proteins and consequently their fate upon triage is also a complete mystery: Specifically, how the crosstalk between the folding and degradation machineries is mediated. An in-depth evaluation of these topics should be addressed through studies of the entire aggregate proteome under normal and proteotoxic stress conditions. The analyses could be facilitated by using quantitative mass-spectrometry approaches and supplementary high-throughput proteomic methods, such as the GPS (Global Protein Stability) approach, developed at the S. Elledge Lab [66]. GPS that has been an excellent platform for a proteome-scale analysis of protein turnover under various physiological and disease conditions, can now be implemented in the investigation of transient-QC bodies and other insoluble protein aggregates.

The more we uncover the mechanism governing transient-QC turnover the better we understand what might turn these into pathological, senile lesions. Hence, a comprehensive biochemical insight of transient-QC biology will advance our ability to diagnose early predisposition to accumulate protein depositions and consequently, to develop therapies to attenuate disease progression.

## References

[1] Alzheimer A. (1906). A uber einen eigenartigen schweren erkrankungsprozess der hirninde. Neurol. Cent..

[2] Hippius H., Neundörfer G. (2003). The discovery of Alzheimer’s disease. Dialogues Clin. Neurosci..

[3] Johnston J.A., Ward C.L., Kopito R.R. (1998). Aggresomes: A cellular response to misfolded proteins. J. Cell Biol..

[4] Kopito R.R. (2000). Aggresomes, inclusion bodies and protein aggregation. Trends Cell Biol..

[5] Hao R., Nanduri P., Rao Y., Panichelli R.S., Ito A., Yoshida M., Yao T.-P. (2013). Proteasomes activate aggresome disassembly and clearance by producing unanchored ubiquitin chains. Mol. Cell.

[6] Kaganovich D., Kopito R., Frydman J. (2008). Misfolded proteins partition between two distinct quality control compartments. Nature.

[7] Miller S.B.M., Ho C.-T., Winkler J., Khokhrina M., Neuner A., Mohamed M.Y.H., Guilbride D.L., Richter K., Lisby M., Schiebel E. (2015). Compartment-specific aggregases direct distinct nuclear and cytoplasmic aggregate deposition. EMBO J..

[8] Escusa-Toret S., Vonk W.I.M., Frydman J. (2013). Spatial sequestration of misfolded proteins by a dynamic chaperone pathway enhances cellular fitness during stress. Nat. Cell Biol..

[9] Sontag E.M., Samant R.S., Frydman J. (2017). Mechanisms and functions of spatial protein quality control. Annu. Rev. Biochem..

[10] Ross C.A., Poirier M.A. (2004). Protein aggregation and neurodegenerative disease. Nat Med..

[11] Fändrich M. (2007). On the structural definition of amyloid fibrils and other polypeptide aggregates. Cell. Mol. Life Sci..

[12] Goedert M. (2015). Neurodegeneration. Alzheimer’s and Parkinson’s diseases: The prion concept in relation to assembled Aβ, tau, and α-synuclein. Science.

[13] Hartl F.U. (2017). Protein misfolding diseases protein misfolding diseases. Annu. Rev. Biochem..

[14] Duggan M., Torkzaban B., Ahooyi T.M., Khalili K., Gordon J. (2020). Age-related neurodegenerative diseases. J. Cell. Physiol..

[15] Malinovska L., Kroschwald S., Munder M.C., Richter D., Alberti S. (2012). Molecular chaperones and stress-inducible protein sorting factors coordinate the spatio-temporal distribution of protein aggregates. Mol. Biol. Cell.

[16] Wooten M.W., Geetha T., Babu J.R., Seibenhener M.L., Peng J., Cox N., Diaz-Meco M.-T., Moscat J. (2008). Essential role of sequestosome 1/p62 in regulating accumulation of Lys63-ubiquitinated proteins. J. Biol. Chem..

[17] Lu K., Psakhye I., Jentsch S. (2014). A new class of ubiquitin-Atg8 receptors involved in selective autophagy and polyQ protein clearance. Autophagy.

[18] Oling D., Eisele F., Kvint K., Nyström T. (2014). Opposing roles of Ubp3-dependent deubiquitination regulate replicative life span and heat resistance. EMBO J..

[19] Wang N., Ma Q., Peng P., Yu Y., Xu S., Wang G., Ying Z., Wang H. (2020). Autophagy and ubiquitin-proteasome system coordinate to regulate the protein quality control of neurodegenerative disease-associated DCTN1. Neurotox. Res..

[20] Sha Z., Schnell H.M., Ruoff K., Goldberg A. (2018). Rapid induction of p62 and GABARAPL1 upon proteasome inhibition promotes survival before autophagy activation. J. Cell Biol..

[21] Limanaqi F., Biagioni F., Gambardella S., Familiari P., Frati A., Fornai F. (2020). Promiscuous roles of autophagy and proteasome in neurodegenerative proteinopathies. Int. J. Mol. Sci..

[22] Kandasamy G., Andréasson C. (2018). Hsp70-Hsp110 chaperones deliver ubiquitin-dependent and -independent substrates to the 26S proteasome for proteolysis in yeast. J. Cell Sci..

[23] Shiber A., Breuer W., Brandeis M., Ravid T. (2013). Ubiquitin conjugation triggers misfolded protein sequestration into quality control foci when Hsp70 chaperone levels are limiting. Mol. Biol. Cell.

[24] Gottesman S., Wickner S., Maurizi M.R. (1997). Protein quality control: Triage by chaperones and proteases. Genes Dev..

[25] Mogk A., Bukau B., Kampinga H.H. (2018). Cellular handling of protein aggregates by disaggregation machines. Mol. Cell.

[26] Lum R., Tkach J.M., Vierling E., Glover J.R. (2004). Evidence for an unfolding/threading mechanism for protein disaggregation by Saccharomyces cerevisiae Hsp104. J. Biol. Chem..

[27] Kaimal J.M., Kandasamy G., Gasser F., Andréasson C. (2017). Coordinated Hsp110 and Hsp104 activities power protein disaggregation in saccharomyces cerevisiae. Mol. Cell. Biol..

[28] Gao X., Carroni M., Nussbaum-Krammer C., Mogk A., Nillegoda N.B., Szlachcic A., Guilbride D.L., Saibil H.R., Mayer M.P., Bukau B. (2015). Human Hsp70 disaggregase reverses parkinson’s-linked α-synuclein amyloid fibrils. Mol. Cell.

[29] Nillegoda N.B., Kirstein J., Szlachcic A., Berynskyy M., Stank A., Stengel F., Arnsburg K., Gao X., Scior A., Aebersold R. (2015). Crucial HSP70 co-chaperone complex unlocks metazoan protein disaggregation. Nature.

[30] Shorter J. (2011). The mammalian disaggregase machinery: Hsp110 synergizes with Hsp70 and Hsp40 to catalyze protein disaggregation and reactivation in a cell-free system. PLoS ONE.

[31] Hoppe T., Branzei D. (2017). Stefan Jentsch (1955–2016)-Maestro of the ubiquitin family. EMBO J..

[32] den Brave F., Cairo L.V., Jagadeesan C., Ruger-Herreros C., Mogk A., Bukau B., Jentsch S. (2020). Chaperone-mediated protein disaggregation triggers proteolytic clearance of intra-nuclear protein inclusions. Cell Rep..

[33] Ho C.T., Grousl T., Shatz O., Jawed A., Ruger-Herreros C., Semmelink M., Zahn R., Richter K., Bukau B., Mogk A. (2019). Cellular sequestrases maintain basal Hsp70 capacity ensuring balanced proteostasis. Nat. Commun..

[34] Berger S.E., Nolte A.M., Kamiya E., Hines J.K. (2020). Three J-proteins impact Hsp104-mediated variant-specific prion elimination: A new critical role for a low. Curr. Genet..

[35] Serlidaki D., van Waarde M.A.W.H., Rohland L., Wentink A.S., Dekker S.L., Kamphuis M.J., Boertien J.M., Brunsting J.F., Nillegoda N.B., Bukau B. (2020). Functional diversity between HSP70 paralogs caused by variable interactions with specific co-chaperones. J. Biol. Chem..

[36] Preston G.M., Guerriero C.J., Metzger M.B., Michaelis S., Brodsky J.L. (2018). Substrate insolubility dictates Hsp104-dependent endoplasmic-reticulum-associated degradation. Mol. Cell.

[37] García-Mata R., Bebök Z., Sorscher E.J., Sztul E.S. (1999). Characterization and dynamics of aggresome formation by a cytosolic GFP-chimera. J. Cell Biol..

[38] Hjerpe R., Bett J.S., Keuss M.J., Solovyova A., McWilliams T.G., Johnson C., Sahu I., Varghese J., Wood N., Wightman M. (2016). UBQLN2 Mediates Autophagy-Independent Protein Aggregate Clearance by the Proteasome. Cell.

[39] Andersson V., Hanzén S., Liu B., Molin M., Nyström T. (2013). Enhancing protein disaggregation restores proteasome activity in aged cells. Aging.

[40] Guo Q., Lehmer C., Martínez-Sánchez A., Rudack T., Beck F., Hartmann H., Pérez-Berlanga M., Frottin F., Hipp M.S., Hartl F.U. (2018). In situ structure of neuronal C9orf72 Poly-GA aggregates reveals proteasome recruitment. Cell.

[41] Bäuerlein F.J.B., Saha I., Mishra A., Kalemanov M., Martínez-Sánchez A., Klein R., Dudanova I., Hipp M.S., Hartl F.U., Baumeister W. (2017). In situ architecture and cellular interactions of PolyQ inclusions. Cell.

[42] Thibaudeau T.A., Anderson R.T., Smith D.M. (2018). A common mechanism of proteasome impairment by neurodegenerative disease-associated oligomers. Nat. Commun..

[43] Cliffe R., Sang J.C., Kundel F., Finley D., Klenerman D., Ye Y. (2019). Filamentous aggregates are fragmented by the proteasome holoenzyme. Cell Rep..

[44] McLoughlin F., Kim M., Marshall R.S., Vierstra R.D., Vierling E. (2019). HSP101 interacts with the proteasome and promotes the clearance of ubiquitylated protein aggregates. Plant Physiol..

[45] Martín-Aparicio E., Yamamoto A., Hernández F., Hen R., Avila J., Lucas J.J. (2001). Proteasomal-dependent aggregate reversal and absence of cell death in a conditional mouse model of Huntington’s disease. J. Neurosci..

[46] Carpenter K., Bell R.B., Yunus J., Amon A., Berchowitz L.E. (2018). Phosphorylation-mediated clearance of amyloid-like assemblies in meiosis. Dev. Cell.

[47] Walters K.J., Kleijnen M.F., Goh A.M., Wagner G., Howley P.M. (2002). Structural studies of the interaction between ubiquitin family proteins and proteasome subunit S5a. Biochemistry.

[48] Kleijnen M.F., Alarcon R.M., Howley P.M. (2003). The ubiquitin-associated domain of hPLIC-2 interacts with the proteasome. Mol. Biol. Cell.

[49] Kleijnen M.F., Shih A.H., Zhou P., Kumar S., Soccio R.E., Kedersha N.L., Gill G., Howley P.M. (2000). The hPLIC proteins may provide a link between the ubiquitination machinery and the proteasome. Mol. Cell.

[50] Funakoshi M., Sasaki T., Nishimoto T., Kobayashi H. (2002). Budding yeast Dsk2p is a polyubiquitin-binding protein that can interact with the proteasome. Proc. Natl. Acad. Sci. USA.

[51] Tsuchiya H., Ohtake F., Arai N., Kaiho A., Yasuda S., Tanaka K., Saeki Y. (2017). In vivo ubiquitin linkage-type analysis reveals that the Cdc48-Rad23/Dsk2 Axis contributes to k48-linked chain specificity of the proteasome. Mol. Cell.

[52] Kim H.T., Goldberg A.L. (2018). UBL domain of Usp14 and other proteins stimulates proteasome activities and protein degradation in cells. Proc. Natl. Acad. Sci. USA.

[53] Munari F., Barracchia C.G., Franchin C., Parolini F., Capaldi S., Romeo A., Bubacco L., Assfalg M., Arrigoni G., D’Onofrio M. (2020). Semisynthetic and enzyme-mediated conjugate preparations illuminate the ubiquitination-dependent aggregation of tau protein. angew. Chem. Int. Ed. Engl..

[54] Morimoto D., Walinda E., Fukada H., Sou Y.-S., Kageyama S., Hoshino M., Fujii T., Tsuchiya H., Saeki Y., Arita K. (2015). The unexpected role of polyubiquitin chains in the formation of fibrillar aggregates. Nat. Commun..

[55] Gavrilov Y., Hagai T., Levy Y. (2015). Nonspecific yet decisive: Ubiquitination can affect the native-state dynamics of the modified protein. Protein Sci..

[56] Baker R.T., Tobias J.W., Varshavsky A. (1992). Ubiquitin-specific proteases of Saccharomyces cerevisiae. Cloning of UBP2 and UBP3, and functional analysis of the UBP gene family. J. Biol. Chem..

[57] Amerik A.Y., Hochstrasser M. (2004). Mechanism and function of deubiquitinating enzymes. Biochim. Biophys Acta.

[58] Lee M.J., Lee B.H., Hanna J., King R.W., Finley D. (2011). Trimming of ubiquitin chains by proteasome-associated deubiquitinating enzymes. Mol. Cell. Proteom..

[59] Mao P., Smerdon M.J. (2010). Yeast deubiquitinase Ubp3 interacts with the 26 S proteasome to facilitate Rad4 degradation. J. Biol. Chem..

[60] Kriegenburg F., Jakopec V., Poulsen E.G., Nielsen S.V., Roguev A., Krogan N., Gordon C., Fleig U., Hartmann-Petersen R. (2014). A chaperone-assisted degradation pathway targets kinetochore proteins to ensure genome stability. PLoS Genet..

[61] De Poot S.A.H., Tian G., Finley D. (2017). Meddling with Fate: The proteasomal deubiquitinating enzymes. J. Mol. Biol..

[62] Baxter B.K., Craig E.A. (1998). Isolation of UBP3, encoding a de-ubiquitinating enzyme, as a multicopy suppressor of a heat-shock mutant strain of S. cerevisiae. Curr. Genet..

[63] Kim Y.E., Hipp M.S., Bracher A., Hayer-Hartl M., Hartl F.U. (2013). Molecular chaperone functions in protein folding and proteostasis. Annu. Rev. Biochem..

[64] Mogk A., Bukau B. (2017). Role of sHsps in organizing cytosolic protein aggregation and disaggregation. Cell Stress Chaperones.

[65] Rosenzweig R., Nillegoda N.B., Mayer M.P., Bukau B. (2019). The Hsp70 chaperone network. Nat. Rev. Mol. Cell Biol..

[66] Yen H.-C.S., Xu Q., Chou D.M., Zhao Z., Elledge S.J. (2008). Global protein stability profiling in mammalian cells. Science.

